# Protein–Polyelectrolyte Interaction: Thermodynamic Analysis Based on the Titration Method †

**DOI:** 10.3390/polym11010082

**Published:** 2019-01-07

**Authors:** Xiaohan Wang, Kai Zheng, Yi Si, Xuhong Guo, Yisheng Xu

**Affiliations:** 1 State-Key Laboratory of Chemical Engineering, East China University of Science and Technology, Shanghai 200237, China; ellery9381@foxmail.com (X.W.); ZKai3824@163.com (K.Z.); guoxuhong@ecust.edu.cn (X.G.); 2 Institute of Vascular Surgery, Fudan University, 180 Fenglin road, Shanghai 200032, China; 3 International Joint Research Center of Green Energy Chemical Engineering, East China University of Science and Technology, Shanghai 200237, China; 4 Engineering Research Center of Xinjiang Bingtuan of Materials Chemical Engineering, Shihezi University, Xinjiang 832000, China

**Keywords:** polyelectrolyte, complexation, electrostatics, thermodynamic analysis, isothermal titration calorimetry

## Abstract

This review discussed the mechanisms including theories and binding stages concerning the protein–polyelectrolyte (PE) interaction, as well as the applications for both complexation and coacervation states of protein–PE pairs. In particular, this review focused on the applications of titration techniques, that is, turbidimetric titration and isothermal titration calorimetry (ITC), in understanding the protein–PE binding process. To be specific, by providing thermodynamic information such as pH_c_, pH_φ_, binding constant, entropy, and enthalpy change, titration techniques could shed light on the binding affinity, binding stoichiometry, and driving force of the protein–PE interaction, which significantly guide the applications by utilization of these interactions. Recent reports concerning interactions between proteins and different types of polyelectrolytes, that is, linear polyelectrolytes and polyelectrolyte modified nanoparticles, are summarized with their binding differences systematically discussed and compared based on the two major titration techniques. We believe this short review could provide valuable insight in the understanding of the structure–property relationship and the design of applied biomedical PE-based systems with optimal performance.

## 1. Introduction

The investigation of the interaction between polyelectrolyte (PE) and proteins remains one of the most important research fields in biotechnology. It is well-established that proteins and PEs could form complexes and coacervates/precipitates at certain conditions, driven by non-specific and non-covalent interactions, primarily electrostatic interactions [1,2,3]. Compared with specific binding between biomolecular pairs with complementary epitopes such as biotin–avidin and antigen–antibody, the protein–PE complexation is a promising prospect in building a multi-functional biomedical scaffold via a flexible and cost-effective approach. In this way, the structure of the protein–PE complex could be tailored by modulating protein–PE interactions, satisfying the needs for various applications such as biosensing [4,5], pharmacology [6,7], protein separation [8,9], and tissue engineering [10]. Furthermore, a thorough understanding of the protein–PE interaction could help identify vascular circulation and toxicology of PE-based biomaterials because they would be exposed to various proteins/biomolecules such as human serum albumin (HSA), trypsin (TRP), and hemo-globin (Hb) of different content in vivo, known as the opsonization effect [11,12]. By modulating key parameters related to protein–PE binding, such as surface charge, hydrophobicity, and chain flexibility, the biocompatibility of the newly developed PE-based materials could be assessed and optimized. 

To understand the protein–PE interaction, different analysis methods, such as dynamic light scattering (DLS) [13], small-angle scattering [14], surface plasmon resonance [15], capillary electrophoresis [16], and microscopy [17], were utilized for characterizing various parameters, such as size, mass, morphology, and adsorption kinetics of the protein–PE complexes. Among them, titration techniques, including turbidimetric titration and isothermal titration calorimetry (ITC), have been gaining increasing significance because of their repeatability, credibility, and sensitivity, as well as the sufficient thermodynamic information they provide. Many researchers have relied on these techniques to obtain qualitative and quantitative information, such as binding stages, binding affinity, stoichiometry, and ultimately the driving forces behind protein–PE binding [18,19,20].

On the basis of the thermodynamic studies on the interaction between proteins and PE for both linear and colloidal, we discussed the mechanisms explored by thermodynamic analysis and possible applications of protein–PE interactions in this short review. In particular, we briefly reviewed two theories significantly promoted by Paul Dubin, that is, charge regulation and charge anisotropy theory, and three binding stages in protein–PE binding, and explored the application of titration techniques, that is, turbidimetric titration and ITC, in understanding the protein–PE binding process. We hope this review can promote the understanding of the protein–PE complexation process and provide guidance for developing applied biomedical PE-based materials with optimal performance. 

## 2. Understanding of Protein–PE Binding Mechanisms

In this section, we discussed the microscopic process behind the protein–PE complexation, as well as analytical methods to characterize these processes. In particular, the application of titration techniques, for example, turbidimetric titration and ITC, in understanding the protein–PE interaction process has been extensively reviewed.

### 2.1. Charge Regulation and Charge Anisotropy

**Figure 1:**
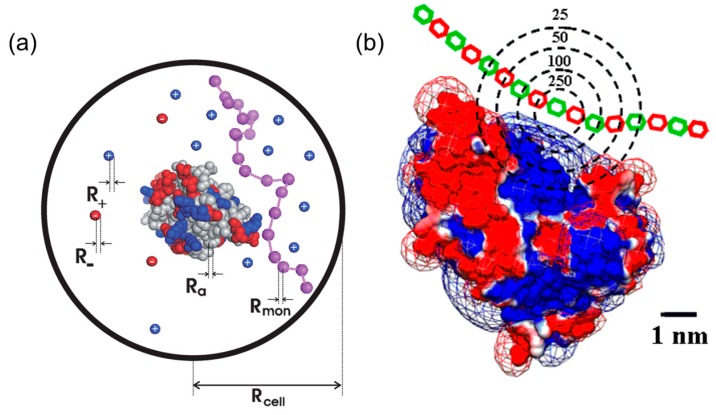
Schematic representation of different protein–PE binding mechanisms. For charge regulation theory (**a**), polyeletrolytes, represented by purple chain, and proteins with positive (blue) and negative (red) residues are surrounded by counter ions within a spherical space denoted as a cell. For charge anisotrpy theory (**b**), proteins with positive (blue) and negative (red) patches as visualized by Delphi interacts with polyelectrolytes with different charged units (red and green). Figure 1 was taken from the works of [21,30].

There are two main proposed mechanisms for protein–PE interactions, that is, charge regulation theory [21,22,23] and charge anisotropy theory [24,25,26,27], both of which could explain some special phenomena occurring in protein–PE binding, such as protein–PE binding “on the wrong side *p*I”. According to charge regulation theory in Figure 1a, the interaction between PE and proteins is confined within limited space. The presence of polyelectrolytes would attract its corresponding counter ions from protein by columbic force so the micro-environment, including local pH and ion distribution of the proteins, was changed by the electrical field of the PEs. Therefore, the protonation states of acidic or basic residues among proteins could be altered, leading to a different charge profile and electrostatic properties in comparison with its normal pattern. For example, under the influence of negative polyelectrolytes, the proteins would be surrounded by more negative counter ions and feature higher local pH, which would render more proteins’ residues positive, even at *p*H > *p*I. Bonsson et al. utilized this theory to analyze different protein–PE binding and proved its effectiveness by Monte-Carlo simulations, especially at lower ionic strength [21]. However, the charge regulation theory failed to explain the ionic strength dependence of PE–protein binding or high selectivity achieved by PE or PE-modified nanoparticles on proteins with similar charge (bovine serum albumin/β-lactoglobulin and β-lactoglobulin isoforms, BLGA/BLGB). For example, Ballauff et al. investigated the interaction between β-lactoglobulin (BLG) and spherical polyelectrolyte brushes (SPB) via ITC combined with statistical model calculations [28]. Judging from positive enthalpy change (Δ*H* > 0) and its significant decrease with increasing ionic strength, they concluded that counter ion release, rather than charge regulation, was the major driving force for BLG–SPB interaction. 

The drawbacks presented above could be well addressed in the charge anisotropy theory proposed by Dubin et al. [26,29,30]. Compared with charge regulation, charge anisotropy theory pays more attention on the internal electrostatic heterogeneity of proteins rather than the external influence from PEs. According to the theory in Figure 1b, different “charge patches”, highly sensitive to conditions such as ionic strength and pH, were locally located on the protein surface. When protein–PE complexation occurs, charge patches with the same and opposite charges both interact with PEs, generating a short-range attraction/long range repulsion (SALR) effect [31]. Therefore, an appropriate amount of salt could always screen long-range repulsions, but preserve short-range attractions, leading to maximal binding affinity at certain ionic strength, and the non-monotonic ionic dependence is commonly observed in many cases for both linear PEs and PE-functionalized nanoparticles [32,33,34]. Moreover, charge patches profiles of proteins with similar *p*I or even structures could be evidently different, such that their phase boundaries of complexation and coacervation/precipitation could diverge with regard to each other. For example, BLGA and BLGB, two variants of BLG, only differ in one amino acid residue in structure, and the replacement of neutral aspartic acid into negative glycine would endow BLGA with a larger negative charge patch and higher binding affinity to linear PEs and charged gold nanoparticles [32,35,36].

Although it is still controversial which mechanism plays a dominant role in the binding process, there is an agreement that both of those mechanisms work in certain circumstances [37]. According to a previous report [38], the charge regulation mechanism predominates for selective protein binding at a lower ionic strength. However, increasing ionic strength eventually raises the dipole moment and the dominant effect turns into the charge patch mechanism, well explained by the charge anisotropy mechanism.

### 2.2. Titration Techniques for Protein–PE Binding Studies

As various analysis methods applied for characterizing protein–PE binding have been extensively discussed in another review by Dubin et al. [39], we mainly focused on titration methods, especially turbidimetric titration and ITC, in this review. Titration approaches serve as effective means of qualitatively identifying the complexation states and quantitatively obtaining thermodynamic parameters such as enthalpy/entropy exchange and binding affinity. Generally, because of their inherent connection, turbidimetric titration and ITC were often applied together to obtain convincing thermodynamic parameters of protein–PE binding. 

#### 2.2.1. Turbidimetric Titration

**Figure 2:**
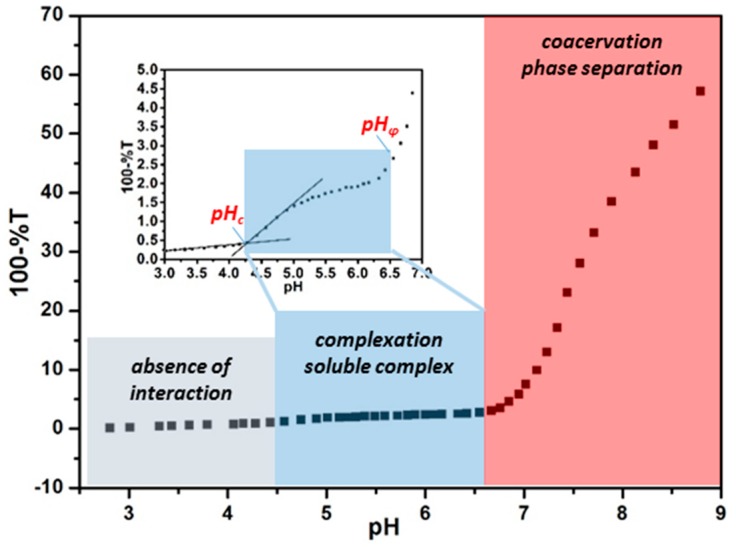
Typical turbidimetric titration curve for bindings between β-lactoglobulin (BLG) and positive charged magnetic nanoparticles at *I* = 5 mM. Grey, blue, and pink areas indicate the absence of interaction, selective complexation, and coacervation stages, respectively. Inset image is the enlarged local version for identification of the second stage, as well as pH_c_**.** The data was taken from the work of [40] and the figure was replotted.

Turbidimetric titration, involving adjusting pH gradually while monitoring the transmittance variation simultaneously, can be utilized to qualitatively characterize the binding pattern between proteins and PEs. During the PE–protein interaction process, intra-particle complexes, inter-particle complexes, coacervates, or precipitates would form successively [25], leading to increased particle size and elevated turbidity. On the basis of the typical turbidimetric titration curve in Figure 2 [40], the binding process could be divided into three stages: absence of interaction, formation of soluble complex, and phase separation of coacervates/precipitates. The critical pH of those three stages can be denoted as pH_c_ and pH_φ_, which represent the onset of complexation and phase separation, respectively. Generally speaking, pH_c_, at which binding energy begins to exceed *kT*, is a semi-quantitative reflection of binding affinity and is an inherent parameter only contingent on protein/PE types at fixed ionic strength [41]. In comparison, pH_φ_, where the net charge of protein–PE system is close to zero, is sensitive to parameters influencing charge stoichiometry such as concentration and molecular weight of PE [42]. According to charge anisotropy theory, by modulating parameters such as ionic strength, proteins with similar *p*I could exhibit different binding pattern, that is, different pH_c_ and pH_φ_, so that they could be discriminated and enriched separately by selective coacervation with PE or nanoparticles [32,43]. 

#### 2.2.2. Isothermal Titration Calorimetry (ITC)

Although turbidimetric titration can provide plentiful information about the protein–PE complexation, it is still necessary to rely on more precise and systematic characterization to cross-validate the data and further understand thermodynamics, such as the binding affinity of the protein–PE interaction. In terms of analyzing protein–PE binding from microscopic view, few characterizations can provide as sufficient thermodynamic parameters as ITC. ITC can directly measure the heat released or absorbed in the binding process and the thermodynamic information can be derived quantitatively from the protein–PE interaction, from which interaction mechanisms, including driving forces, affinity, and stoichiometry, could be explored.

**Figure 3:**
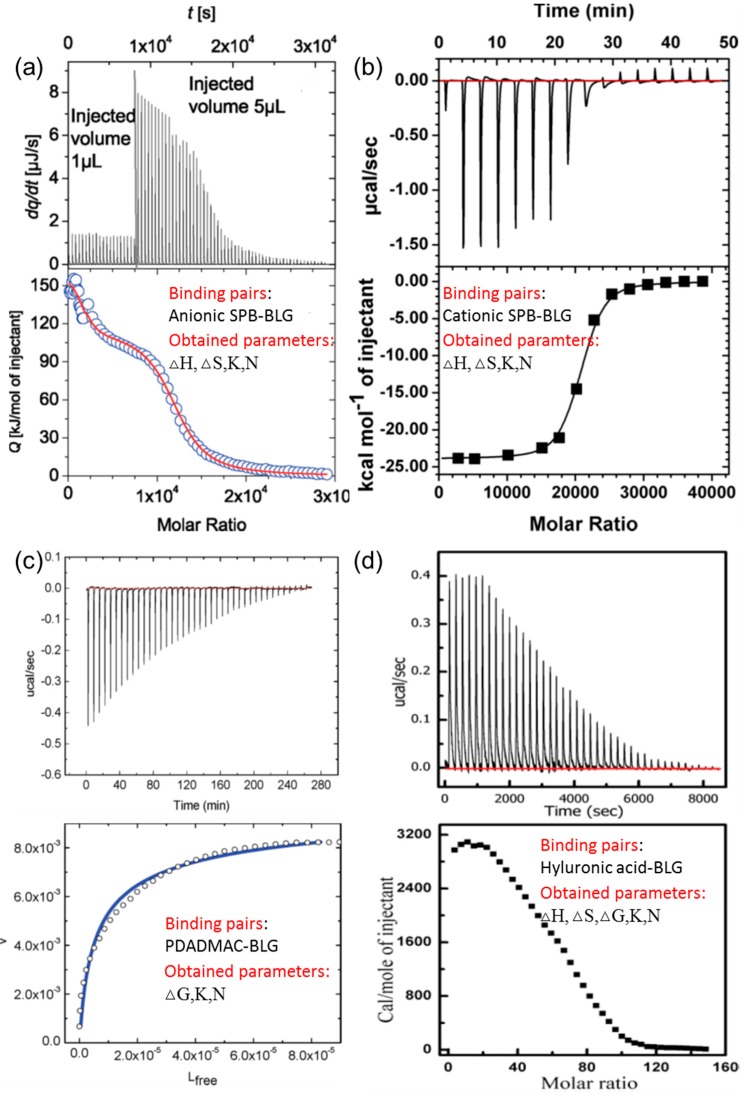
Typical isothermal titration calorimetry (ITC) data for binding of BLG with (**a**) anionic spherical polyelectrolyte brushes (SPB), (**b**) cationic SPB, (**c**) poly(dimethyldiallylammonium chloride)(PDADMAC), and (**d**) hyaluronic acid. Figure 3 was taken from the works of [28,30,32,47].

In a typical ITC curve, the vertical peaks represent the heat change in the sample cell at each syringe injection, with the syringe and cell containing PE or protein, respectively. The enthalpy change, △*H*, could be calculated from heat integration of the first injection and binding constant, *K*_b_, could be obtained by fitting the binding isotherm according to the appropriate model. For example, the independent one-site model, based on the assumption that all binding sites are identical and each binding features the same △*H*, was commonly used for analyzing isotherms of the protein–PE interaction, while two-site or multiple binding models would be a suitable choice for complicated protein–PE interaction situations such as bindings incorporating aggregation, denaturation, and configuration transformation of proteins [44,45,46]. Moreover, according to the formula △*G* = −*RT*ln*K*_b_ and △*G* = △*H* − *T*△*S*, entropy change, △*S*, another important indicator of the driving force of binding, could be derived. The typical ITC original curves for different types of PE substrates, as well as corresponding fitted binding isotherms, are presented in Figure 3.

From the analysis of those thermodynamic parameters of △*H* and △*S*, the possible driving force of the protein–PE interaction could be clearly identified. Generally, the protein–PE binding was enthalpy driven (△*H* < 0) or entropy driven (△*S* > 0), which corresponds to different biomolecular interactions predominating the binding process [48]. The non-covalent interaction processes, including electrostatic interaction, hydrophobic interaction, and hydrogen bonding between PE chains and protein domains are commonly recognized as negative enthalpy and entropy change (△*H* < 0, △*S* < 0), as flexible ligands and domains within PE and proteins are constrained to form complexation. In comparison, the desolvation process, including reorganization and release of water molecules as well as counter ions, is endothermic with positive entropy change (△*H* > 0, △*S* > 0) because of the energy needed to destroy the original structure confining ion and water molecules. The non-covalent binding and desolvation occur simultaneously and the overall binding could be regarded as the combination of those two processes, as shown in the following equations [49]

(1) PE+Protein⇌PE−Protein

(2) $${\mathrm{xH}}_{2}O_{N}+{\mathrm{yH}}_{2}O_{B}⇌\left(x+y-z\right)H_{2}O_{N-B}+{\mathrm{zH}}_{2}O$$

(3) $$\mathrm{PE}\cdot {\mathrm{xH}}_{2}O_{N}+\mathrm{Protein}\cdot {\mathrm{yH}}_{2}O_{B}⇌\mathrm{PE}-\mathrm{Protein}\cdot \left(x+y-z\right)H_{2}O_{N-B}+{\mathrm{zH}}_{2}O$$

where H_2_O_B_, H_2_O_N_, H_2_O_N-B_ refer to water molecules associated with protein, PE, and the protein–PE complex, respectively.

Therefore, whether the binding between PE and protein is endothermic or exothermic depends on which of the processes mentioned above predominates during the overall complexation process. In the majority of PE–protein binding cases, electrostatic interaction plays a major role, so the binding processes are commonly of enthalpy origin with △*H* < 0, while entropy-driven processes with △*S* > 0 prevails in some cases for positively charged nanoparticles [36,50,51]. It is noteworthy that the reversal of enthalpy could occur even for PE–protein pairs with similar structures. For example, researchers have prepared the same types of anionic spherical polyelectrolyte brushes featuring polystyrene (PS) core grafted with poly(styrene sulfonate) (PSS) and poly(acrylic acid) (PAA), respectively. According to ITC data on BLG–SPB binding, opposite heat change signals and enthalpy change could be observed, which means that after binding with BLG, the PS–PSS brushes feature positive enthalpy and entropy change, while the exact opposite happens to PS–PAA brushes. This interesting phenomenon could be attributed to different charge polarity of the grafted polyelectrolyte chains, which could contribute to additional non-covalent interaction such as hydrophobic interaction and hydrogen bonds [28,52]. Xu et al. utilized ITC to study BLG–poly(dimethyldiallylammonium chloride)(PDADMAC) bindings at different conditions, and the binding reaction transferred from exothermic to endothermic as the titrates changed from BLG to BLGA/B [32]. In this case, the higher pH of titration for BLGA/B may play an important role because, at pH close to pH_φ_, aggregation becomes more evident, leading to a greater extent of water reorganization and release. In our current study (not reported yet), reversed heat change from exothermic to endothermic binding after increasing pH from 4.5 to 7.5 was observed for binding between proteins and cationic polyelectrolyte modified magnetic nanoparticles, which could be attributed to separate binding stages predominated by different processes, as mentioned above. Although it is rather difficult to specify the individual contribution of non-covalent binding and desolvation into precise proportions, ITC is still an essential characterization approach for PE–protein binding in terms of identifying the driving force of binding and providing guidance for designing favorable binding processes.

**Figure 4:**
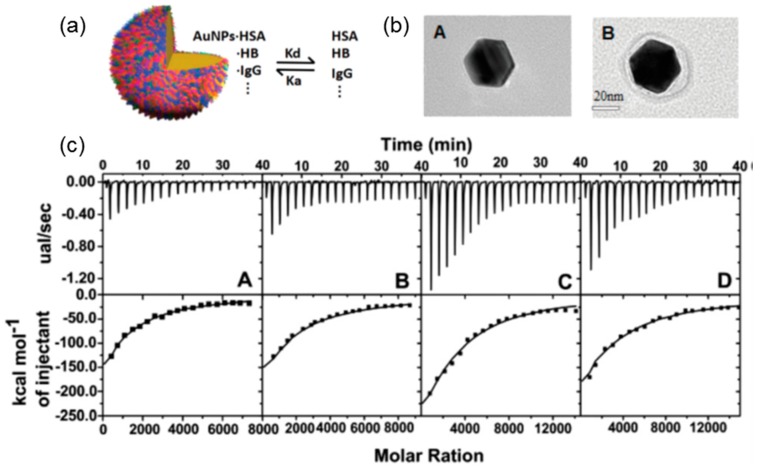
(**a**) The schematic representation of plasma proteins’ adsorption and dissociation on an AuNP (**b**). The typical TEM image of a single AuNP (left) and its complex with plasma protein (right) (**c**). AuNP size dependence of ITC titration with plasma protein at 18 °C. From left to right, the average size of AuNP increased from 15 nm to 25 nm, 40 nm, and 70 nm. Figure 4 was taken and modified from the work of [54]. HAS—human serum albumin; HB—hemo-globin.

Besides revealing the driving force of binding, ITC could also provide valuable information about binding affinity. As mentioned above, turbidimetric titration could help acquire qualitative identification of binding affinity, that is, pH_c_. However, as pH_c_ is determined with slight increase of turbidity (0.1%–0.5% in %T) compared with a non-zero slope, uncertainties may be caused by instrument drift or artificial errors. Serving as an effective, non-destructive, in-situ measurement tool of binding, ITC could present binding affinity and binding stoichiometry directly based on the measured binding heat and the chosen model. Systematic studies have been constructed on PE–protein binding affinity via ITC to achieve enhanced selectivity for protein purification or to gain an in-depth understanding of protein corona formation within living organisms [43,46,53]. For example, Dubin et al. utilized synthesized PEs PDADMAC [32] and natural PEs [30], as well as hyaluronic acid (HA), respectively, to study their selective binding with BSA and BLG, two types of proteins with similar *p*I (BSA: ~4.9; BLG: ~5.1). Derived from combinatorial studies of turbidimetric titrations and ITC in Table 1, they found that BLG and BSA exhibits higher affinity to positively charged PDADMAC and negatively charged hyaluronic acid, respectively, which could be attributed to the concentrated negative (BLG) and positive (BSA) charge patch for those two proteins. Zhang et al. explored the adsorption behaviors of various serum proteins on gold nanoparticles (AuNPs) with different sizes via ITC (Figure 4) [54]. Combined with dynamic light scattering and fluorescence quenching results, they found that particles with larger sizes and proteins with more surface cysteine residues tend to exhibit higher binding affinity. In a word, thermodynamic results provided by ITC could serve as the foundation to understand protein–PE interactions in various cases.

## 3. Thermodynamic Studies of the Protein–PE Interaction 

To study the protein–PE interaction, two types of PEs, that is, linear PEs and PE-modified nanoparticles, are mainly used for different contexts. For linear PEs, the certain and tunable structures could provide valuable insights into designing desirable binding, while for charged nanoparticles, the environmental and biological behaviors could be predicted for real application situations [55,56], especially for biomedical applications, in which they would be exposed to various proteins within human bodies [46,57,58]. Moreover, based on the thermodynamic studies, both linear PEs and PE-modified nanoparticles could be developed for various applications, which will be discussed in the next section.

### 3.1. Linear Polyelectrolytes

**Figure 5:**
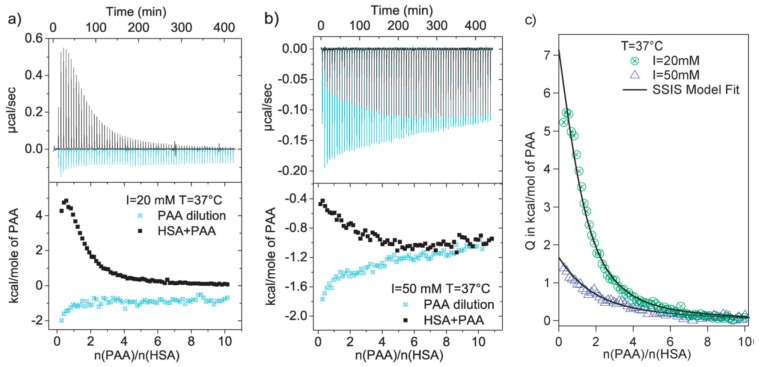
(**a**) ITC data of adsorption of poly(acrylic acid) (PAA) upon HSA and the corresponding heats of dilution of PAA at pH = 7.2, *T* = 37.1 °C, and (**a**) *I* = 20 mM and (**b**) *I* = 50 mM. (**c**) Binding isotherm corrected for the heat of dilution at 37.1 °C, and *I* = 20 mM and 50 mM. Figure 5 was taken from the work of [59].

The interaction between linear PEs has been extensively investigated and could serve as the foundation for studying the interaction of proteins with PE-modified nanoparticles, because the colloidal or metal core-shell nanoparticles typically compromise an organic or inorganic core accompanied by numerous end-grafted linear PEs and, for most circumstances, it is the surface PE coatings rather than core materials that interact with proteins [48]. Dubin and Ballauff et al. utilized a series of synthetic or natural charged linear PEs, such as heparin [27], hyaluronic acid [30], PAA [25], and PDADMAC [32], to study their interactions with various proteins via both titration techniques and model simulations. For example, Antonov et al. studied the critical conditions of complexation and coacervation for PDADMAC–BSA pairs via turbidimetric titration, and found that the coacervation state could be well-tuned between entering and exiting by pH, ionic strength, and stoichiometry. Yu et al. conducted comprehensive studies on the binding behaviors of human serum albumin with poly(acrylic acid) occurring “on the wrong side of *p*I” [59]. On the basis of the strong positive enthalpy and entropy change derived from ITC (Figure 5), they attribute the origin of the binding process to heterogeneous distribution of protein charge, which could lead to significant counter ion release during binding reflected by the dramatic entropy increase. Moreover, the binding energy change, △*G*, obtained from ITC, correlated well with the results of coarse-grained Langevin computer simulations.

### 3.2. Polyelectrolytes Modified Nanoparticles

A broad range of charged nanoparticles, including metal nanoparticles and colloidal nanoparticles, has been developed for various applications, especially biomedical applications, to achieve enhanced therapeutic efficacy [60,61,62]. Therefore, these nanoparticles can interact with a vast range of biomolecules, especially plasma proteins [63,64], during its circulation in vivo. Moreover, nanoparticle–protein assemblies can serve as flexible scaffolds for various biomedical applications such as drug delivery [65] and biosensing [66]. 

**Figure 6:**
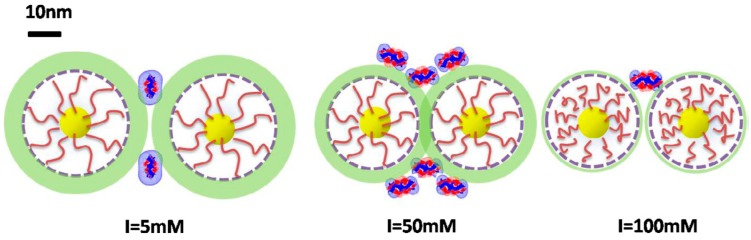
Schematic illustration of BLG/Fe_3_O_4_–PMATAC nanoparticles interaction at various ionic strengths. Green shading area represents a potential contour. The scale bar was added to define 10 nm length. Figure 6 was taken from the work of [34].

Compared with linear PEs, PE-modified nanoparticles feature much higher charge density, and hence higher binding affinity and likely higher selectivity toward proteins. Moreover, the PE chains grafted to nanoparticles are more rigid and less flexible than its free counterparts in aqueous solution, so spatial constraints mutually exerted by both nanoparticles and proteins have to be considered in addition to the interplay between different surface functionalities. For example, positive PE-modified magnetic nanoparticles were prepared and their binding affinity toward proteins, characterized by turbidimetric titration and ITC, was enhanced at higher ionic strengths because of their closer inter-particle distance caused by screened mutual repulsion (Figure 6) [34]. The calculated surface potential of proteins conformed well to the binding constant derived from ITC, providing convincing evidence for the proposed mechanism. In addition, the same dependence was also confirmed by Wang’s research through ultra-precise thermal analysis of ITC, in which subtle differences in heat change caused by hydrophobic groups from magnetic nanoparticles were observed with a non-monotonic dependence of binding affinity on ionic strength [40]. 

**Figure 7:**
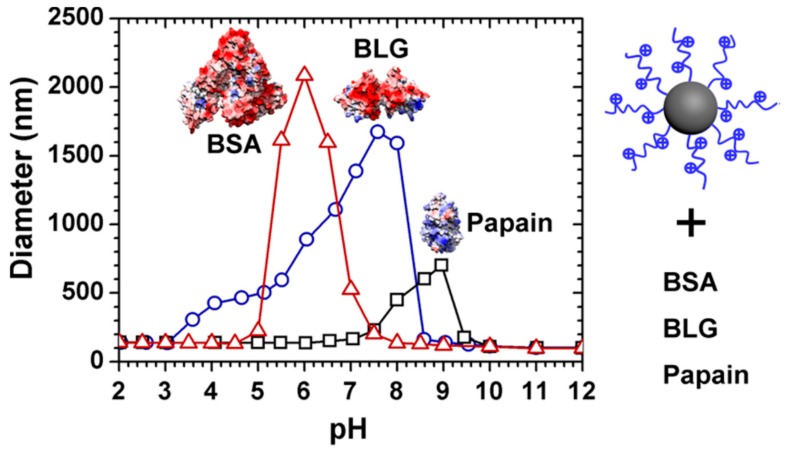
Typical turbidimetric titration curves representing different binding stages of SPB, as well as its selective binding toward different proteins. Figure 7 was taken from the work of [47].

Among various PE-modified nanoparticles, spherical polyelectrolyte brushes have been widely used in proteins because of their high loading capacity, stable micro-environment, and tunable performance. Ballauff et al. firstly used SPB as nanocarriers for proteins and studied their interaction via DLS, small angle X-ray scattering (SAXS), and ITC supplemented by model simulation [14,28,67]. Based on ITC analysis, binding “on the wrong side of *p*I” between protein and SPB could be clearly observed and could be attributed to the entropy-driven process because of counter ion release [28,68]. However, a single type of SPBs, mostly anionic SPBs, and proteins were used in their cases, which lacked the comparison between different protein–SPB bindings to study the effect of molecular structures of SPBs on binding patterns. Subsequently, various brushes, both anionic and cationic, and proteins including BSA, BLG, and papain were used to study their phase behavior [47]. Interestingly, turbidimetric titration curves revealed that weak polyelectrolyte such as PAA or cationic poly(2-aminoethylmethacrylate hydrochloride) (PAEMH) modified SPBs could exhibit separated binding, aggregation, and releasing region in full pH window (Figure 7), which was caused by the pH-sensitive charge profiles for both SPBs and proteins [47,69]. Within the whole pH range, the charges of SPBs and proteins are opposite to each other in aggregation stages, while in the other two stages, they carried the same overall charge. Moreover, based on ITC analysis, those SPBs feature different binding affinity towards proteins depending on protein types. Generally, anionic SPBs bind more strongly to proteins with more positive patches, and vice versa [70]. It is noteworthy that “quenched” brushes, that is, strong polyelectrolyte modified brushes, typically do not exhibit pH-induced aggregation and release caused by the lack of pH-responsiveness of brush layers [71,72,73], so SPBs grafted by weak PEs are more promising for protein separation and purification purposes. 

**Figure 8:**
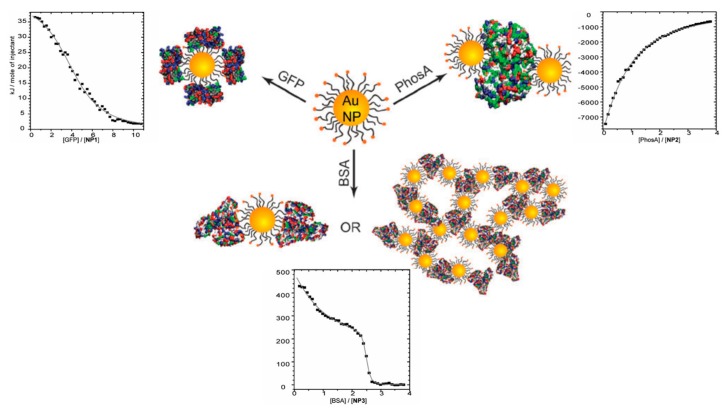
Binding conformations and corresponding ITC curves of charged gold nanoparticles with green fluorescence protein (GFP), acid phosphatase (PhosA), and BSA. Figure 8 was taken from the work of [48].

In addition to selective binding and loading of proteins, the studies of nanoparticle–protein binding is of vital importance to their biomedical applications, because nanoparticles would preferably interact with plasma proteins such as serum albumin, and the corona around nanoparticles would ultimately affect its biological fate during circulation [74]. Gold nanoparticles are most commonly used because of their well-defined and tunable surface composition and structure [75,76], and ITC was observed to be able to understand the binding structures between proteins and nanoparticles. Although most of the related research focused on ligands rather than PE-modified nanoparticles, the highly charged nature of those two types of particles still shows a certain degree of similarity when binding with proteins. De et al. studied the interaction between positive gold nanoparticles with different proteins including green fluorescence protein (GFP), acid phosphatase (PhosA), and BSA [47]. Based on the ITC data shown in Figure 8, they concluded that relative size could dictate their binding profiles. Moreover, the thermodynamic quantities (△*H/T*△*S*) obtained by ITC exhibited a linear relationship, revealing the resemblance between nanoparticle–protein and protein–protein bindings.

## 4. Thermodynamic Studies Guiding for Protein–PE Applications

On the basis of different binding states discriminated by titration, the protein–PE interaction could be applied for different purposes. In general, the formation of a soluble protein–PE complex could endow the system with the capability to immobilize proteins for various purposes, while the coacervate or precipitate state could be utilized for protein purification by selective phase separation. To develop novel PE-based advanced materials, thermodynamic studies could provide guidance for optimizing performance for their application.

### 4.1. Protein Immobilization

As mentioned above, turbidimetric titration could help identify three different stages for protein–PE bindings, and the intermediate complexation state could be harvested to immobilize and stabilize proteins because it could prevent protein from further aggregation via electrostatic repulsion. For example, Wang et al. observed a large range of plateau during turbidimetric titration between SPB and BLG, which could be attributed to the repulsion between complexes with the same charge [47]. Both synthesized and natural PEs have been used for protein stabilization and the prevention of aggregation could be clearly verified by turbidimetric titration curves, as the turbidity will stop increasing drastically once aggregation was inhibited. For instance, Xu et al. utilized heparin to reverse and inhibit the aggregation of three types of proteins, including BSA, BLG, and Zn-insulin, while keeping the original protein structure [27]. According to turbidimetric titration and DLS results, the aggregation of those proteins, in both the native and denatured state, could be well-controlled by forming soluble complexes with heparin. 

As the PEs could provide proteins with a stable micro-environment and help preserve their normal functions in the second binding stages, enzymes have great potential to be incorporated into PE-based systems for various catalytic situations. At the same time, ITC can be applied to evaluate the loading capability by measuring binding constant and stoichiometry. For example, Xu et al. loaded amyloglucosidase into the brush layers of magnetic spherical polyeletrolyte brushes. According to ITC results, strong binding between enzymes and SPB could be clearly observed even on the “wrong side” of binding. In this way, enhanced enzymatic activity and magnetic recyclability could be achieved simultaneously [51].

**Figure 9:**
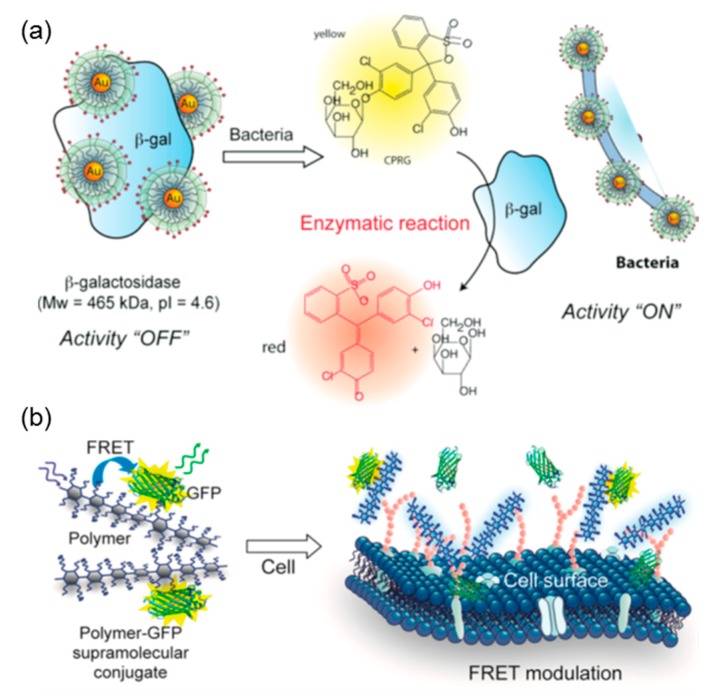
Schematic illustration of “chemical nose/tongue” sensing practice based on the electrostatically driven complexation of catalytic (**a**) and fluorescent (**b**) proteins. Figure 9 was taken from the works of [66,80].

Moreover, PEs and charged nanoparticles could interact differentially with different proteins during complexation stages and this difference in binding affinity, as revealed by ITC, could be utilized to generate fluorescent [77], colorimetric [65], or even fragrant [78] read-out signals using array-based PEs and functional proteins. Rotello et al. conducted extensive research in terms of this field. Generally, after forming electrostatically driven complexation with fluorescent proteins or enzymes such as β-galactosidase (β-Gal), the positively charged gold nanoparticles [79] or linear polyelectrolytes [80] could inhibit their functionality temporarily, while the presence of analytes could disturb the binding equilibrium and the restored functionality of proteins could contribute to different read-out signals according to analyte types (Figure 9). In this process, ITC plays a vital role in screening proteins and PEs with appropriate binding affinity, because binding that is too strong would prevent functional proteins from releasing and weak binding would lead to instability of the sensing conjugates. By choosing the appropriate functional protein and series of charged sensing agent with the help of ITC, reliable array-based signals could be obtained. After processing the read-out signals, researchers successfully discriminated and identified different proteins [79] and bacteria [81], as well as metastistic [82], cancerous [60], and normal cells [83]. Moreover, these novel biosensors based on protein complexation have been developed for many real-life applications, such as testing strips for drinking water [84] and diagnosis of liver fibrosis [85].

### 4.2. Protein Purification

**Figure 10:**
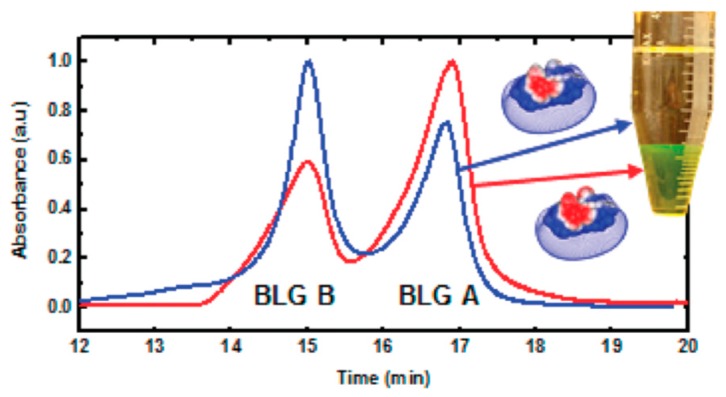
Ion exchange chromatography analysis of BLG-A and -B composition in different phases after PE coacervation of native BLG (A/B = 1:1). Red line, coacervate; blue line, supernatant. Figure 10 was taken from the work of [32].

PEs could be utilized to obtain target proteins from their mixtures by forming coacervates selectively under certain conditions. According to charge anisotropy theory, proteins feature a heterogeneous surface dotted with different charge patches and hydrophobic domains. Therefore, non-specific interactions, including electrostatic and hydrophobic interactions of Pes, have the potential to achieve selective binding and phase separation with proteins. In fact, a certain degree of selectivity toward proteins has been achieved by both linear PE chains [32] and PE-modified colloidal particles [47,69]. Based on turbidimetric titration and isothermal titration results, positive PE chains and particles exhibit evidently stronger binding affinity toward proteins with more negatively charge patches, such as BLG versus BSA and BLGA versus BLGB. Furthermore, the selectivity, represented by ΔpH_c_ and ΔpH_φ_, could be modulated by changing ionic strength. By choosing the appropriate condition with the largest ΔpH_φ_ and forming phase separation between PDADMAC and mixed proteins (BSA&BLG, *w*/*w* = 1:1), the supernatant and coacervates feature totally different composition with relative purity increased to 90%. Moreover, the variants of BLG, BLG-A, and BLG-B could be separately condensed at the coacervate and supernatant phase after phase separation between native BLG and PDADMAC (shown in Figure 10). The purification efficiency could be even higher using polyampholytic polypeptide [52]. The PE could be recycled by ultrafiltration or precipitation by adding a co-solvent like alcohols. Charged gold nanoparticles with different hydrophobicity have also been reported to exhibit selectivity toward proteins with similar structures [35]. Therefore, the recycling of the purification agent could be more convenient by functionalization such as introducing magnetic targetablity into the system, which is currently under investigation by our group.

## 5. Conclusions and Outlook

In this review, we have described recent developments on the understanding of protein–PE interactions using titration methods, which provide guidance for various application purposes with an in-depth understanding of the interactions. The applications of titration techniques of turbidimetric titration and ITC in protein–PE binding studies have been systematically reviewed. On the basis of thermodynamic studies on the binding between proteins and PE, both linear chains and nanoparticles, the protein–PE interaction could be utilized for various biomedical applications such as protein immobilization and protein purification. 

As structural uncertainties and heterogeneities still prevail in complicated PE systems, as well as in proteins, a thorough understanding of the structure–property relationship still remains a challenge. In addition, although various types of PEs and proteins have been studied thermodynamically, the experimental conditions, such as stoichiometry, pH, and ionic strength, varied greatly, leading to fragmented ideas and great difficulties in establishing general rules and theories. To address these problems, binding interfaces with well-defined and controllable structures are required and a relatively uniform standard for protein–PE binding characterizations should be established so that different studies could be compared and combined. Moreover, the current titration techniques still suffer from some drawbacks, such as dependence on model selection and accurate mole concentration calculation. Therefore, they need to be cross-validated by other complementary characterizations and computational simulations. 

Currently, most of the studied protein–PE complexation and coacervation are electrostatically driven and the effecte of other non-specific interactions, such as hydrophobic interaction and hydrogen bonds, are still unclear. Therefore, further studies in this area could focus on the verification of the relative contributions of separation interactions, which are still rather challenging at the moment. The traditional protein–PE models could be also expanded to more complicated systems such as drugs, enzymes, and antibodies for proteins. In addition, more research attention should be paid to PE-modified nanoparticles, because the nanoparticle–protein interaction studies nowadays mainly focus on monolayer protected nanoparticles and spherical polyelectrolyte brushes. The accumulation of PE chains on nanoparticles has great potential to enlarge the discrimination in binding with proteins, endowing the system with an improved application prospect. In a word, despite the current drawbacks and limits, with deepening understanding of the structure–property relationship, protein–PE interaction could be applied to further develop biomedical PE-based materials with smart and optimal performance.

## Figures and Tables

**Table 1:** Thermodynamic properties obtained from the independent site-binding model for polymer−protein interactions. Conditions for poly(dimethyldiallylammonium chloride) (PDADMAC)/protein and hyaluronic acid (HA)/protein are pH 5.3, I = 100 mM and pH 4.3, I = 100 mM, respectively. BLG—β-lactoglobulin. The data was taken from the works of [30,32].

Polymer/Protein	*N*	*K*_obs_ (M^−1^)	△*H* (kcal/mol)	*T*△*S* (cal/mol)
HA/BSA	38 ± 1	389 ± 31	4.77 ± 0.01	8.30 ± 0.01
PDADMAC/BSA	80 ± 2	740 ± 30	−4.15 ± 0.02	−0.26 ± 0.03
HA/BLG	51 ± 1	228 ± 22	2.97 ± 0.01	6.18 ± 0.02
PDADMAC/BLG	50 ± 1	1900 ± 340	−4.67 ± 0.02	−0.2 ± 0.1
